# A New Evolutionary Algorithm-Based Home Monitoring Device for Parkinson’s Dyskinesia

**DOI:** 10.1007/s10916-017-0811-7

**Published:** 2017-09-25

**Authors:** Michael A. Lones, Jane E. Alty, Jeremy Cosgrove, Philippa Duggan-Carter, Stuart Jamieson, Rebecca F. Naylor, Andrew J. Turner, Stephen L. Smith

**Affiliations:** 1 School of Mathematical and Computer Sciences, Heriot-Watt University, Edinburgh, UK; 2 Department of Neurology, Leeds General Infirmary, Leeds, UK; 3 Department of Electronic Engineering, University of York, York, UK

**Keywords:** Parkinson’s disease, Dyskinesia, Home monitoring, Genetic programming

## Abstract

Parkinson’s disease (PD) is a neurodegenerative movement disorder. Although there is no cure, symptomatic treatments are available and can significantly improve quality of life. The motor, or movement, features of PD are caused by reduced production of the neurotransmitter dopamine. Dopamine deficiency is most often treated using dopamine replacement therapy. However, this therapy can itself lead to further motor abnormalities referred to as dyskinesia. Dyskinesia consists of involuntary jerking movements and muscle spasms, which can often be violent. To minimise dyskinesia, it is necessary to accurately titrate the amount of medication given and monitor a patient’s movements. In this paper, we describe a new home monitoring device that allows dyskinesia to be measured as a patient goes about their daily activities, providing information that can assist clinicians when making changes to medication regimens. The device uses a predictive model of dyskinesia that was trained by an evolutionary algorithm, and achieves AUC>0.9 when discriminating clinically significant dyskinesia.

## Introduction

Parkinson’s Disease (PD) is a common, progressive neurodegenerative condition affecting approximately 1% of those over 65 years of age. The motor features of PD are caused by degeneration of dopamine-producing neurones in the basal ganglia. A lack of dopamine leads to a number of motor abnormalities, including slowed movements with smaller amplitude (bradykinesia), stiffness and tremor. Motor features of PD can be treated through the use of dopamine replacement drugs, such as levodopa. Over time, prolonged exposure to dopamine replacement therapy, combined with the neurodegenerative changes of PD, can lead to dyskinesia: involuntary jerking and spasms of the muscles that can affect the whole body, and can be very severe. Dyskinesia is common; for example, it affects 90% of patients with PD treated with levodopa after ten years [2]. Despite this, the exact pathophysiological basis of dyskinesia is unknown [26].

To reduce the risk of dyskinesia, clinicians attempt to administer drugs at the lowest dose required to treat the motor features of PD. As PD worsens over time, the dose required to treat the motor features increases. The risk of inducing dyskinesia, or making it more severe and more prolonged in someone who already has it, is increased if too high a dose is prescribed. Most patients see their clinician relatively infrequently, and medication changes only tend to occur at the time of review. Additionally, studies have shown that people with PD are often unaware of when they have dyskinesia [28] and that patient-completed symptom diaries tend to lack accuracy [10]. This means it is very difficult for clinicians to know when in the day dyskinesia is occurring, motivating the need for automated methods of monitoring dyskinesia that may be used in the patient’s own home.

In this paper, we describe a new home monitoring device that uses a predictive model to identify episodes of dyskinesia from accelerometry time series data. Predictive models are computational or mathematical models that can predict the characteristics of previously unseen data [13, 18]. [1] review previous work on applying these approaches to movement data collected from PD patients, and [22] give a more general account of the use of wearable devices for objectively assessing PD motor symptoms. A number of previous studies have considered methods for identifying dyskinesia in movement data [3, 5, 11, 16, 23, 27]; a common aspect is the use of spectral features to characterise dyskinesia [5, 16, 23, 27], focusing on the low frequency bands where dyskinetic activity generally occurs. The main difference in our work lies in the use of raw acceleration data to build predictive models. This provides more information for the training algorithm to work with. It also makes relatively few assumptions about the physiological appearance of dyskinesia, which is important given the limited understanding of this movement [26]. Since our algorithm uses symbolic mathematical expressions to characterise movements in the time domain, it is able to discriminate movements based on their overall shape, rather than their frequencies [19, 20], which is useful for discriminating dyskinesia from other low-frequency movements.

## Materials and methods

### Devices

**Fig. 1:**
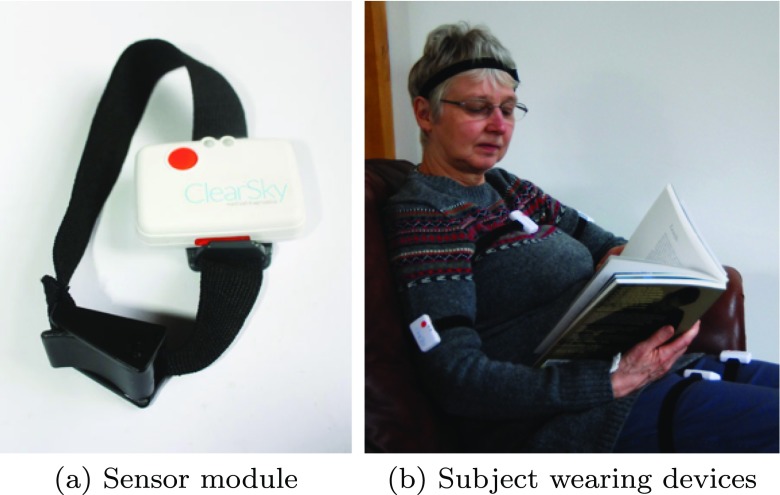
Six degrees of freedom wearable accelerometer and gyroscope sensor modules used in this study

To measure a patient’s movements, we used sensing modules comprising a tri-axial accelerometer and tri-axial gryoscope (see Fig. 1). The modules have a sample rate of 100Hz, and are able to store data in local memory. In the clinical studies, this data was downloaded to a computer for further processing following each recording session. In the final version of the product, it is anticipated that the data will be intermittently broadcast to an Android mobile phone, where the data will be input to the predictive model and the resulting measures of dyskinetic activity broadcast to the patient’s clinician. The sensing modules are lightweight and wireless, allowing for patients to move freely and perform their usual daily activities. They were fitted to each patient’s legs, arms, torso, head and trunk using adjustable bands.

During the clinical study periods, we also made use of an infrared video camera, which recorded footage of each patient during the time their movements were being measured. This allowed associations to be made between the accelerometry data and the patient’s activities when labelling periods of movement in the data set.

### Clinical study data

Twenty three PD patients were involved in two clinical studies: the first involved 6 patients, and the second involved 17. Table 1 gives the demographics of the two study groups. The patients in both groups were broadly similar, although the initial group of patients were on average slightly older with more severe motor and dyskinesia scores. The movements of each patient were recorded continuously over a period of hours: 6 for the first group, 2 for the second group. Patients were recorded sequentially, and all patients within a single study wore the same set of sensor modules. However, the two studies used different sets of sensor modules, and this provides a useful source of diversity when assessing the generality of the predictive models.

**Table 1:** Summary of the two clinical studies

	Study 1	Study 2
Number of patients	6	17
Assessment period (hours)	6	2
Gender male:female	4:2	11:6
Age (years)	71 ±8.9	65 ±7.3
Disease duration (years)	9.8 ±3.7	8.1 ±3.6
MDS-UPDRS motor score	31 ±19.1	28 ±18.0
PDYS-26 quality of life score	37.6 ±29.2	34.7 ±24.5

To be included in the study, patients required an established diagnosis of PD according to internationally recognised criteria [14] and had to have displayed evidence of dyskinesia during a hospital outpatient clinic consultation performed by a Consultant Neurologist with a specialist interest in movement disorders (SJ, JA). Those who did not meet these criteria, or those with PD and cognitive impairment sufficient enough to prevent their ability to provide informed consent, were excluded. Patients completed demographic details and the 26-item Parkinson Disease Dyskinesia Scale (PDYS-26), a patient-completed measure of the impact of dyskinesia on quality of life (range 0–104, higher numbers indicate greater impact) [15]. The sensors were attached and the video camera was activated. Every hour, motor signs were assessed using the MDS Unified Parkinson’s Disease Rating Scale (MDS-UPDRS) motor examination (range 0–132, higher numbers indicate greater impairment) [9]. Between times, patients were free to move around the unit, although were asked to remain in view of the video camera. No changes were made to medication regimens and so patients were predominately assessed in the ON state. The application of the sensors was not related to the timing of medications. Some patients with unpredictable ON/OFF fluctuations demonstrated both medication states during the recording period. All patients gave informed consent and both studies were granted ethical approval by the National Research Ethics Service (IRAS 84044) and local Research and Development approval from Leeds Teaching Hospitals NHS Trust. The study was added to the NIHR Clinical Research Network (CRN) portfolio (ID 11762).

Following data collection, the video footage was watched by three trained clinicians (JA, JC, PDC), who marked up segments of the video and graded the intensity of dyskinesia in each of the assessed body parts according to Part 3 of the Unified Dyskinesia Rating Scale (UDysRS) [8], which grades dyskinesia from 0 (no dyskinesia) to 4 (incapacitating dyskinesia which prohibits some postures and voluntary movements). Where raters disagreed on the severity of the dyskinesia, the average grade was taken.

Table 2 summarises the data collected during the two studies. Data from the first study was used to compare different classifier models and the utility of different data sources, and then to select a promising classifier from amongst all the trained instances for use in the deployed system. Data from the second study was used to give an unbiased estimate of the generality of this selected classifier on the wider population of patients and sensors.

**Table 2:** Number of examples of each dyskinesia grade collected from the two studies

UDysRS	Study 1	Study 2
0	2933	1747
1	1227	971
2	1688	562
3	681	183
4	64	361

### Genetic programming

Evolutionary algorithms (EAs) are a family of general purpose optimisation algorithms, or *metaheuristics*, that are motivated by biological evolution [6]. They are known to work well in large complex search spaces, which makes them well suited to biomedical problems. EAs carry out a series of moves (known as *mutation* and *crossover*) through a solution search space by making changes to a group of existing candidate solutions that are held in a data structure called a population. EAs are iterative: they begin with a population of random solutions, and at each iteration (or *generation*), a new population is created by applying moves to a subset of the previous generation’s population. This subset is chosen by a selection mechanism, which selects existing solutions in proportion to their objective value (or *fitness*).

Genetic programming (GP) is an EA that optimises executable expressions [24]. In this work, we use GP to find a mathematical expression that describes the relationship between one or more input variables and a target variable. The input variables, in this case, are the input accelerometry values within a window of time series data. The target variable is the degree of dyskinesia that is associated with the time series window; hence, GP is used to find mathematical expressions that describe patterns of acceleration that are indicative of dyskinesia. There are various kinds of GP [24]. In this work, we use Cartesian GP, where an expression is laid out as a grid of functional elements. Cartesian GP has been shown to be competitive against other GP systems [21]. We use a particular variant called implicit context representation Cartesian GP (IRCGP), which uses a low-level representation that is designed to improve the effectiveness of crossover [4].

### Classifier models

**Fig. 2:**
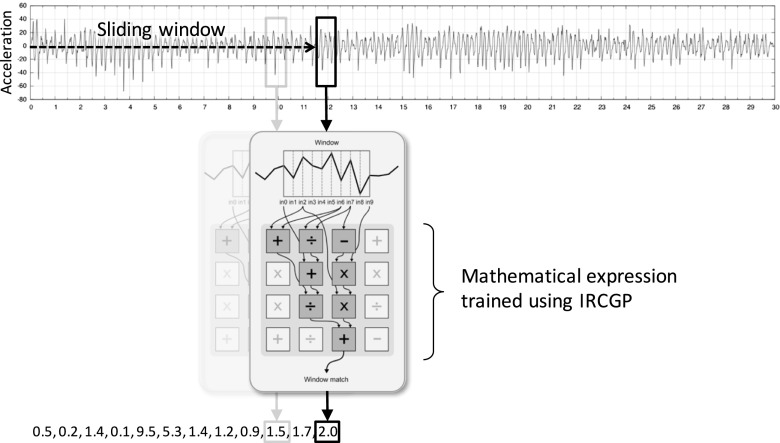
Illustration of how a symbolic mathematical expression trained using IRCGP is used to classify an acceleration time series, showing how the expression is applied to inputs taken from a sliding window. The final classification is the mean of the outputs from each window

IRCGP was used to train a symbolic mathematical expression comprised of up to 36 function instances, selected from the set {+,−,×,÷,mean,min,max,abs}, laid out on a 6 ×6 Cartesian plane and taking inputs from 32 terminal nodes fed from the accelerometry data (see Fig. 2). We used a population size of 200, a generation limit of 100, and standard mutation and crossover probabilities [19].

For each data item, a univariate time series was first created by calculating the magnitude of acceleration at each time index. During classification, a sliding window of length 32 (0.32*s*) is slid along a time series, and the classifier generates an output for each of the *L* − 31 overlapping windows, where *L* is the length of the time series. The classification for the time series is then taken to be the mean of these values, i.e. the mean occurrence of the movement pattern which is described by the symbolic mathematical expression. By applying a threshold to the output range of the classifer, a particular data item can then be classified as either dyskinetic or not dyskinetic.

For comparative purposes, we also trained long-term and short-term spectral classifiers. For the long-term model, the mathematical expression operates on spectral densities from 32 equally spaced intervals in the frequency range 0–50Hz, calculated using the method described in [12]. For the short-term spectral classifier, the expression is applied independently to the spectral densities in each time series window, and then averaged. To make the results directly comparable, these models were formulated such that the evolutionary algorithm searches a space of equal size and dimensionality for each classifier model.

## Results and analysis

All classifiers were assessed using the area under the receiver operating characteristic (ROC) curve, or AUC, when discriminating samples of Grade 3 and 4 dyskinesia from movement samples with no dyskinesia. AUC can be interpreted as the probability that a randomly drawn sample will be allocated to the correct category [17]. In general, we have found that it is easier to generate robust classifiers when Grades 1 and 2 are not used during training. This is possibly due to difficulty in clinically differentiating between these grades of dyskinesia using the UDysRS.

**Fig. 3:**
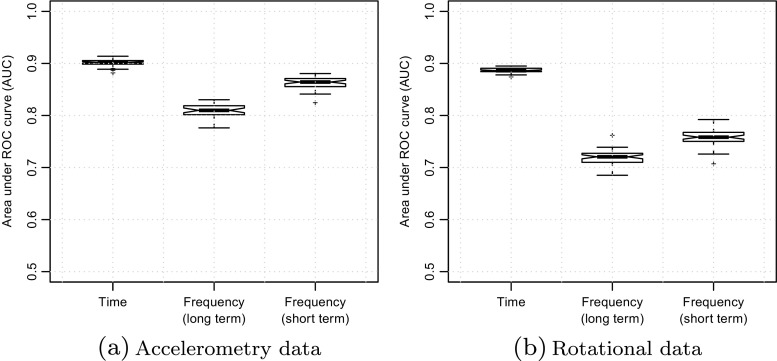
Discriminative ability of classifiers trained to recognise Grade 3 and 4 dyskinesia, comparing the predictive ability of time domain and spectral domain classifiers and the utility of accelerometry and rotational (gyroscopic) data. Notched box plots show distributions of AUC on the test set over 50 independent runs of IRCGP

Figure 3 compares the different classifier models. It can be seen that time domain classifiers have significantly higher AUCs than spectral classifiers, reaching AUCs in excess of 0.9 on the test set. Figure 3 also compares the utility of accelerometry and rotational gryroscope data when training classifiers, showing that more accurate classifiers can be built from accelerometry data. These results suggest that it is easier to find significant discriminatory patterns in the time domain than in the spectral domain, and that patterns may be more evident in planar movements rather than rotational movements.

**Fig. 4:**
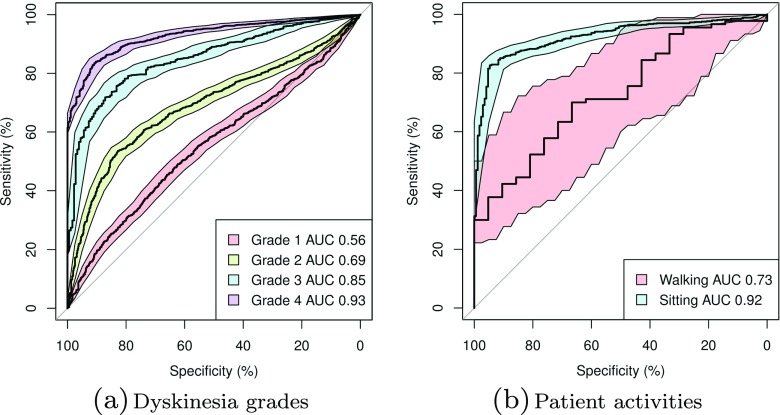
ROC curves for the selected dyskinesia classifier on the second study data set, showing discriminative ability (**a**) for the different UDysRS dyskinesia grades, (**b**) when the patient is carrying out different kinds of activity. Coloured regions show confidence boundaries determined through repeated re-sampling

**Fig. 5:**
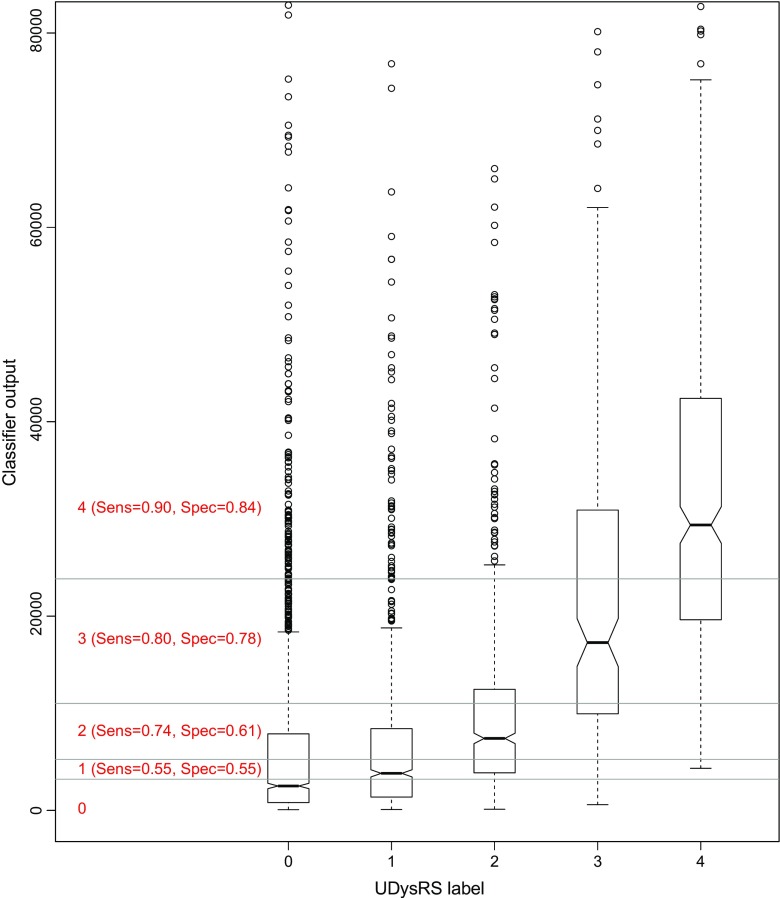
Mapping between dyskinesia grade and classifier outputs on the second study data. Horizontal grey lines show the optimal thresholds in the classifier’s output range for differentiating between classes, and the red text gives the corresponding values of sensitivity and specificity for each clinical grade of dyskinesia

**Fig. 6:**
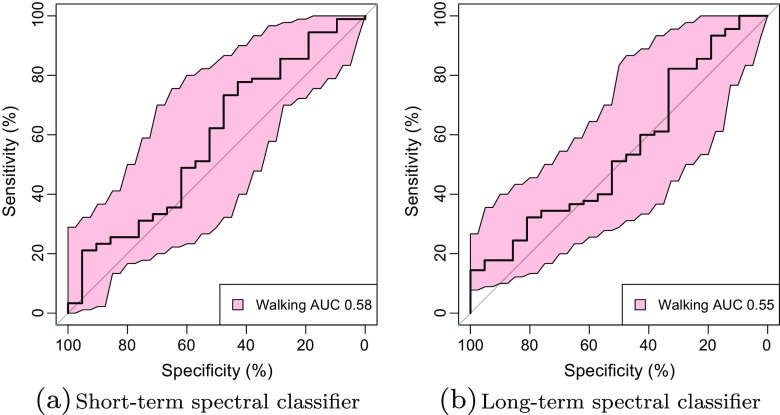
Ability of spectral classifiers with highest test set score to discriminate dyskinesia whilst walking

The classifier instance with the highest test set AUC was selected for further study. To obtain a more robust measure of generality, it was re-evaluated using the movement samples in the second data set. Figure 4a shows the resulting ROC curves, illustrating that the classifier retains a good ability to separate samples with Grades 3 and 4 dyskinesia from samples with no dyskinesia, and has not overfitted the first data set. Figure 5 shows the actual mapping between labelled samples and classifier outputs, indicating that the outputs for the higher dyskinesia grades are clearly separated from those for samples with no dyskinesia. There is some overlap, but this is mostly due to a long tail of outliers in the non-dyskinesia samples. This reflects the observation, also seen in other studies, that certain voluntary movements are very difficult to discriminate from dyskinesia. As an example of this, Fig. 4b compares the discriminative ability of the classifier when a patient is sitting or walking. Walking, in this case, is a low frequency activity, and degrades the accuracy of the classifier. Nevertheless, as Fig. 6 shows, the classifier performs a lot better than the spectral domain classifiers in this respect, indicating the benefit of working in the time domain when discriminating activities of similar spectral frequencies.

**Fig. 7:**
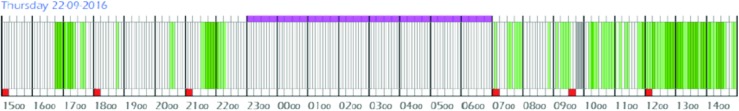
An example of output generated by the system, showing a summary chart for a patient measured over a 24 hour period. The red dots represent the time at which medication was taken, the dark green lines the occurrence of severe dyskinesia (grade 4) and the light green lines, significant dyskinesia (grade 3). The purple line indicates when the patient is asleep

The selected classifier has since been integrated into a tool for clinical use, and Fig. 7 shows an example of the output generated when using the tool to evaluate a *de novo* patient who was not included in the two studies. This summary chart is intended to give clinicians a quick overview of a patient’s response to levodopa over a period of 24 hours, showing when they are experiencing clinically significant degrees of dyskinesia. In this example, the patient experiences two hours of Grade 3 or Grade 4 dyskinesia between 12:00 and 14:00, which might suggest to the clinician that the levodopa dose given at 12:00 is too great and requires reduction.

**Fig. 8:**
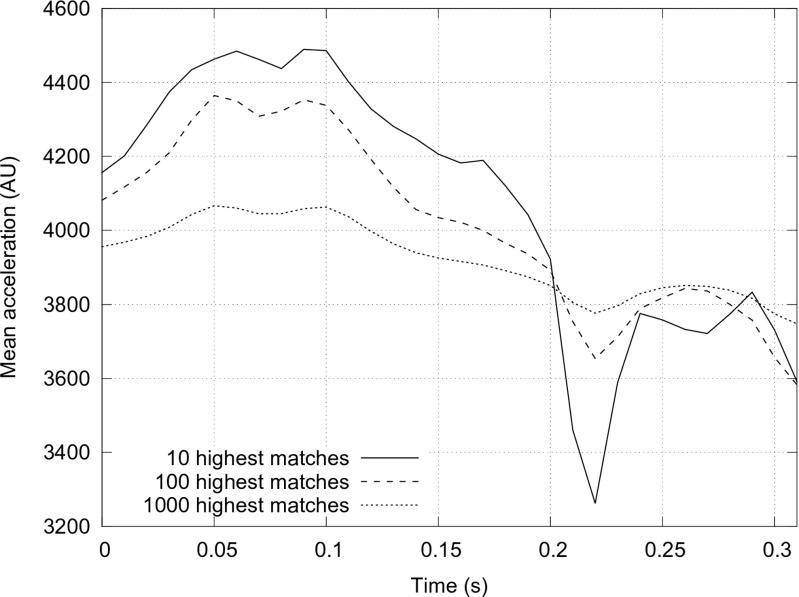
Visualisation of the acceleration pattern recognised by the best performing dyskinesia classifier. Mean acceleration patterns are shown for the 10, 100 and 1000 time series windows that generated the highest outputs

In addition to direct use as a clinical tool, the classifiers can also be used to improve understanding of dyskinesia. In particular, it is possible to visualise the discriminatory pattern of acceleration that is being recognised by the classifier. As an example of this, Fig. 8 plots the means of windows of data which are assigned a particularly high value by the classifier, i.e. periods of movement that the classifier identifies as being particularly dyskinetic. It indicates a pattern comprising two periods of rising then falling acceleration, with the second period of rise and fall being shorter than the first. There is also a prominent dip at the top of the first peak, forming a caldera shape. Whilst the meaning of this pattern is currently unclear, it is well conserved across the data samples, suggesting that it is a significant discriminator of dyskinesia from other movements.

## Conclusions

In this paper, we have described our work on developing a wearable home monitoring system for assessing dyskinesia in Parkinson’s disease patients. The results demonstrate the ability of the system to reliably detect clinically significant dyskinesia, thus providing the information required by clinicians to adjust a patient’s medication and more effectively manage the troublesome side-effects that currently reduce the quality of life of many patients. Our system also has the potential to significantly reduce the clinical costs of managing Parkinson’s disease, estimated to be around $12000 per patient in the year following diagnosis [25], and with the potential to save over £84 million per annum in England alone [7].
